# Importance of Suitable Reference Gene Selection for Quantitative Real-Time PCR: Special Reference to Mouse Myocardial Infarction Studies

**DOI:** 10.1371/journal.pone.0023793

**Published:** 2011-08-17

**Authors:** Bert R. Everaert, Gaëlle A. Boulet, Jean-Pierre Timmermans, Christiaan J. Vrints

**Affiliations:** 1 Laboratory of Cell Biology and Histology, University of Antwerp, Antwerp, Belgium; 2 Laboratory of Cellular and Molecular Cardiology, Antwerp University Hospital, Antwerp, Belgium; Temasek Life Sciences Laboratory, Singapore

## Abstract

**Background:**

Quantitative real-time PCR (qPCR) is a widely used technique for gene expression analysis. Its reliability is highly dependent upon selection of the appropriate reference genes for accurate gene expression normalization. In this study, we investigated the expression stability of 10 commonly used reference genes in a mouse myocardial infarction model.

**Methods & Results:**

The expression stability of the 10 reference genes (Actb, B2m, Eef1a1, Gapdh, Hprt, Polr2a, Ppia, Rpl13a, Tbp, Tpt1) was analyzed using the geNorm software. Overall, the combination of Hprt, Rpl13a and Tpt1 was the most stable reference gene set in our experiments. Gapdh, Polr2a and Actb consistently showed the highest gene expression variability and the expression levels of Gapdh, Polr2a, Actb, B2m and Eef1a1 were found to be selectively up- or downregulated after myocardial infarction. We normalized the expression of Nppb and Vcam1, using different reference gene strategies and demonstrated that their induction after myocardial infarction was most clearly revealed with the optimal reference gene combination. However, the use of suboptimal reference gene combinations resulted in detrimental effects on gene expression levels and variability with a gradual loss of the expression differences and a significant reduction in statistical power.

**Conclusions:**

Hprt, Rpl13a and Tpt1 are a set of stably expressed reference genes for accurate gene expression normalization in myocardial infarction studies in mice. We found that Gapdh, Polr2a and Actb display high expression variability in mouse myocardial infarction tissues and that loss of statistical power and increase in sample size are the evident consequences of choosing suboptimal combinations of reference genes. We furthermore caution against the use of Gapdh, Polr2a, Actb, B2m and Eef1a1 for gene expression normalization in myocardial infarction studies because of selective up- or downregulation after myocardial infarction, which could potentially lead to biased study outcomes.

## Introduction

Quantitative real-time PCR (qPCR) is the method of choice for gene expression research and analysis of biological pathways for several reasons: the technique is fast, extremely sensitive, highly reproducible and can be integrated in high-throughput systems. Therefore, it is routinely applied in many research areas. Despite the advantages and its apparent simplicity, qPCR expression analysis also has a number of caveats, for example the need for a proper endogenous control to normalize relative gene expression data. Although many studies have warned against the use of a single reference gene for gene expression normalization [1], [2], the expression of reference genes, such as glyceraldehyde-3-phosphate dehydrogenase (*Gapdh)* or beta*-*actin *(Actb)*, or the amount of 18S ribosomal RNA (rRNA) are still frequently used as a single endogenous control [3]. However, the use of a single unvalidated reference gene may give rise to biased study results, especially when study conditions are changed or experimental variability is increased [4]. The increase in reference gene variability becomes even more problematic if genes with relatively small expression differences are studied.

In the setting of myocardial infarction and congestive heart failure (CHF), this instability in reference gene expression has already been described. For instance, Brattelid et al. [5] reported that the reduced expression of *Gapdh* compared to 18S rRNA significantly influenced the outcome and interpretation of mRNA expression levels. In another *in vitro* reference gene study, the expression levels of either beta-2 microglobulin (*B2m), Gapdh*, *Actb*, or 18S rRNA were found to be highly dependent upon the experimental conditions [4]. This implicated that the choice for a given reference gene for gene expression normalization could potentially bias relative mRNA expression results and alter the study outcome. These problems on the validity of single reference genes for qPCR gene expression normalization led Vandesompele et al. to propose an innovative strategy by using the geometric mean of an optimal number of stably expressed reference genes (geNorm algorithm) [2]. This approach has subsequently been used by numerous researchers for the optimization of reference gene normalization strategies in distinct research applications.

Thus, stable reference gene expression is a prerequisite for conducting accurate and valid gene expression studies. This is particularly true when reference gene variability is expected to be high, as in the case of myocardial infarction or CHF studies. This study aimed to determine an optimal combination of stably expressed reference genes for use in mouse myocardial infarction studies using the geNorm algorithm. In addition, the effects of using suboptimal combinations of reference genes for gene expression analysis on statistical parameters such as significance, power and sample size were studied.

## Methods

### Ethics Statement

National and European principles of laboratory animal care were followed. The Animal Care and Use Committee of the University of Antwerp approved all animal experimental procedures (Permit Number: 2008-03).

### Selection of candidate reference genes

The selection of the reference genes was based on a literature search on the subject of reference gene studies in myocardial infarction. Five commonly used reference genes were included: *Actb*, *B2m*, hypoxanthine phosphoribosyltransferase 1 (*Hprt*), *Gapdh*, TATA box binding protein (*Tbp*). Furthermore, five less known reference gene candidates were selected based on their promising stability characteristics, as determined by previous reference gene studies: eukaryotic translation elongation factor 1 alpha 1 (*Eef1a1*) [6], polymerase (RNA) II (DNA directed) polypeptide A (*Polr2a*) [7], peptidylprolyl isomerase A (cyclophilin A) (*Ppia*) [8], ribosomal protein L13a (*Rpl13a*) [6], tumor protein, translationally-controlled 1 (*Tpt1*) [6]. Care was taken not to include genes of the same functional pathway, as such avoiding problems of co-regulation and false positive reference gene selection.

### Myocardial infarction mouse model and sample collection

The tissue specimens analyzed in this study were taken from a large mouse myocardial infarction tissue sample set. Myocardial infarction was induced by ligation of the left anterior coronary artery (LAD). In brief, 3- to 6-month-old male mice of C57BL/6 background were were anesthetized (tribromoethanol 0.25mg/g, intraperitoneally), intubated using a 22G intravenous catheter and mechanically ventilated with a small rodent ventilator (MiniVent type 845, Harvard Apparatus, ventilation at 10 µl/g, 180 breaths/min, 2 cm H_2_O positive end-expiratory pressure). A left parasternal thoracotomy was performed transecting ribs 4 and 5. After adequate exposure of the heart, the pericardium was cleaved and the LAD was ligated approximately 2 mm below the left atrial appendage using 7-0 polypropylene sutures (Pronova® BV-1, Ethicon, Johnson & Johnson). Ten age-matched C57BL/6 mice were sham-operated, i.e., without tightening of the LAD ligature. White discoloration of the myocardium, elevation of the ST segment on electrocardiographical monitoring and visual identification of the ligated artery in the infarction zone evidenced successful LAD ligation. Subsequently, the operation wound was closed in layers, mice were weaned from ventilation, extubated and placed under a heat source until full recovery. The tissue sampling was performed one week post-myocardial infarction. Infarcted left ventricle (ILV, n = 10, group 1) was harvested by macrodissection, immediately flash-frozen in liquid nitrogen and stored at −80°C for later use. Likewise, tissue specimens from non-infarcted left ventricular (NILV) free wall zones in myocardial infarction (NILV, n = 10, group 2) and sham-operated mice (sham, n = 10, group 3) were collected and stored. Group characteristics are summarized in Table S1.

### Tissue homogenization, RNA extraction and quality, cDNA synthesis

The tissues were homogenized with an OmniTH tissue homogenizer (Mettler-Toledo) on ice. RNA was isolated using AllPrep DNA/RNA/Protein mini kit (Qiagen) and turbo DNA free kit (Ambion) was used to remove contaminating DNA leftovers, both following the manufacturer's instructions. The RNA concentration and purity were analyzed using a Nanodrop spectrophotometer (Nanodrop technologies), measuring spectral absorption at 260 and 280 nm. The assessment of the RNA integrity was done using Agilent 2100 Bioanalyzer. cDNA was synthesized using the transcriptor first strand cDNA synthesis kit (Roche) according to the manufacturer's instructions, using a combined oligo(dT)/random hexamer primer strategy for reverse transcription (RT). RT was performed on 0.5 µg RNA starting material in a final reaction volume of 20 µl. The RT reaction sequence consisted of incubation at 25°C for 10 minutes, followed by 30 minutes at 55°C. The transcriptor reverse transcriptase enzyme was inactivated by heating to 85°C for 5 minutes. cDNA samples were placed on ice and stored at −20°C until further use.

### qPCR

Taqman® gene expression assays (Applied Biosystems) were used for qPCR analysis on a LightCycler® 480 instrument (Roche) (table 1). All primers were designed to be intron-spanning to prevent the replication of residual contaminating DNA. To minimize the influence of PCR inhibitors in qPCR applications, all cDNA samples were diluted by a factor 50. qPCR was performed on 5 µl cDNA using the LightCycler® 480 Probes Master (Roche) in a final reaction volume of 20 µl in LightCycler® 480 white 96 Multiwell Plates (Roche). All samples were run in duplicate and no template controls (NTC) were included in all runs to exclude possible DNA contamination. Reactions were carried out as follows: after an initial denaturation-activation step at 95°C for 10 min, amplifications consisted of 45 cycles of denaturation at 95°C for 10 s, annealing at 60°C for 30 s and measurement of fluorescence at 72°C for 1 s. The quantification cycle (Cq) was measured using the baseline-independent second derivative maximum method [9]. The assay efficiency was measured by serial dilution (5-point 4-fold dilution series, using triplicates) of cDNA of pooled samples based on the slope of the standard dilution curve (E = 10^(1/-slope)^-1) (table 2).

**Table 1:** Gene abbreviations and respective protein function.

Gene	Full name	Protein function
*Actb*	beta-actin	Cytoskeletal structure protein
*B2m*	beta-2-microglobulin	Beta–chain of MHC class I molecules
*Eef1a1*	eukaryotic translation elongation factor 1 alpha 1	Aminoacyl-tRNA binding to ribosomes during protein biosynthesis
*Gapdh*	glyceraldehyde-3-phosphate dehydrogenase	Oxido-reductase in glycolysis and gluconeogenesis
*Hprt*	hypoxanthine phosphoribosyltransferase 1	Purine synthesis through the purine salvage pathway
*Polr2a*	polymerase (RNA) II (DNA directed) polypeptide A	DNA-dependent RNA polymerase
*Ppia*	peptidylprolyl isomerase A (cyclophilin A)	Protein folding
*Rpl13a*	ribosomal protein L13a	Structural component of the large 60S ribosomal subunit
*Tbp*	TATA box binding protein	RNA polymerase II transcription factor
*Tpt1*	tumor protein, translationally-controlled 1	Involved in calcium binding and microtubule stabilization
*Nppb*	natriuretic peptide B	Cardiac hormone, key role in natriuresis and vasorelaxation
*Vcam1*	vascular cell adhesion molecule 1	Adhesion molecule expressed on inflamed endothelium

**Table 2:** qPCR assay information.

Gene	AB ID	GenBank ID	Amplicon (bp)	E(%)
*Actb*	Mm01205647_g1	NM_007393.3	72	95
*B2m*	Mm00437762_m1	NM_009735.3	77	92
*Eef1a1*	Mm01966109_u1	NM_010106.2	150	88
*Gapdh*	Mm99999915_g1	NM_008084.2	107	94
*Hprt*	Mm00446968_m1	NM_013556.2	65	97
*Polr2a*	Mm00839493_m1	NM_009089.2	85	92
*Ppia*	Mm02342430_g1	NM_008907.1	148	102
*Rpl13a*	Mm01612986_gH	NM_009438.4	122	93
*Tbp*	Mm00446973_m1	NM_013684.3	73	94
*Tpt1*	Mm03009502_g1	NM_009429.2	150	91
*Nppb*	Mm01255770_g1	NM_008726.4	68	94
*Vcam1*	Mm00449197_m1	NM_011693.3	97	93

AB ID: Applied biosystems Taqman® gene expression assay ID; bp: number of base pairs; E: Assay efficiency. The assay efficiency was measured by serial dilution of cDNA of pooled samples based on the slope of the standard dilution curve (E = 10^(1/-slope)^-1) and expressed as percentage (%).

### Reference gene expression stability and statistical analysis

The geNorm software [2] for Microsoft Excel® was used for the analysis of the relative gene expression data. To calculate the expression stability of a given gene (gene stability measure M), the program uses an algorithm based on the mean of the pairwise variation of a given reference gene compared to all other control genes. The higher the value of M, the higher the expression variability of the corresponding reference gene. The least stable gene, i.e., the gene with the highest M value, is excluded from the subsequent analysis and all M values are then recalculated until the two most stable genes remain. A stability plot shows the stepwise exclusion of the most unstable reference gene. To determine the optimal number of reference genes in a particular experimental setting, a pairwise variation coefficient V_n/n+1_ is calculated between two sequential normalization factors NF_n_ and NF_n+1_. Values below a given threshold, signify sufficient reference gene set stability and hence validity for gene normalization. As a general rule, values of ≤0.5 for M and of ≤0.15 for V_n/n+1_ are indicative of a stably expressed reference gene or gene set, respectively [2]. It is recommended to use at least the 3 most stable reference genes. Additionally, using the BestKeeper software tool [10], we performed a coefficient of variance analysis in which the coefficient of variance is expressed as a percentage of the average Cq level. To highlight the effects of the different normalization strategies on statistical parameters, such as significance, power and sample size, the expression of natriuretic peptide B (*Nppb*) and vascular cell adhesion molecule 1 (*Vcam1*) was measured and normalized using different normalization strategies. A non-parametric Mann-Whitney U test was used because of the non-normality of the relative expression data. A two-sided p-value of <0.05 indicated statistical significance. Statistical analysis was performed in PASW® statistics 18 (IBM Corp.). After logarithmic (log_2_) transformation, the statistical power of a two-sided Student's t-test and the sample size necessary to obtain a power of at least 0.8 were calculated using the distribution parameters of the normalized relative expression data [11].

### MIQE guidelines

To increase the reliability and integrity of study results and to promote the effort for experimental consistency and transparency between research laboratories, we adhere to the Minimum Information for Publication of Quantitative Real-Time PCR Experiments (MIQE) guidelines [12]. A MIQE checklist is published as Table S2.

## Results

Thirty samples of left ventricular tissue were harvested. To yield representative results for gene expression normalization in myocardial infarction experiments in mice, both ILV (n = 10, group 1) and NILV (n = 10, group 2) specimens from infarcted hearts, as well as tissue samples of the left ventricular free wall of sham-operated animals (n = 10, group 3) were included in the analysis (supplementary table 1). RNA quantity and quality assessment confirmed good RNA yields and adequate RNA purity and integrity with a mean A_260_/A_280_ ratio of 2.03±0.10 and mean RNA integrity number of 8.06±1.30 respectively.

**Figure 1:**
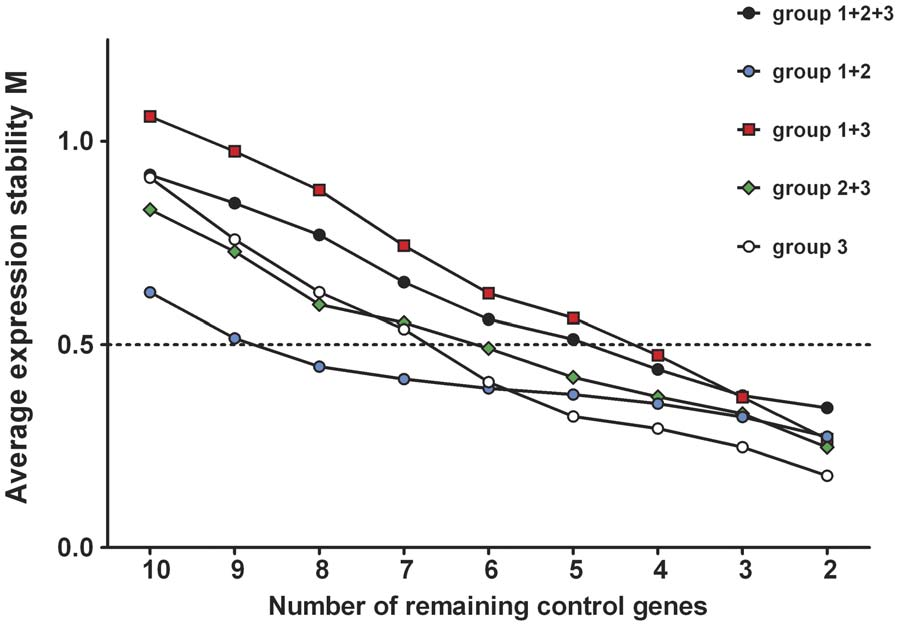
Average expression stability values of reference genes during stepwise exclusion. The average expression stability values for mouse myocardial infarction tissue are depicted for all (n = 30) and different subsets of samples. Pairwise variation decreases from left to right, due to stepwise exclusion of the least stable reference gene. The least stable gene is found on the right; the best pairwise combination is depicted on the left. To retrieve the corresponding gene names, we refer to table 3. Group 1: infarcted left ventricle; group 2: non-infarcted left ventricle: group 3: sham left ventricular heart tissue.

**Figure 2:**
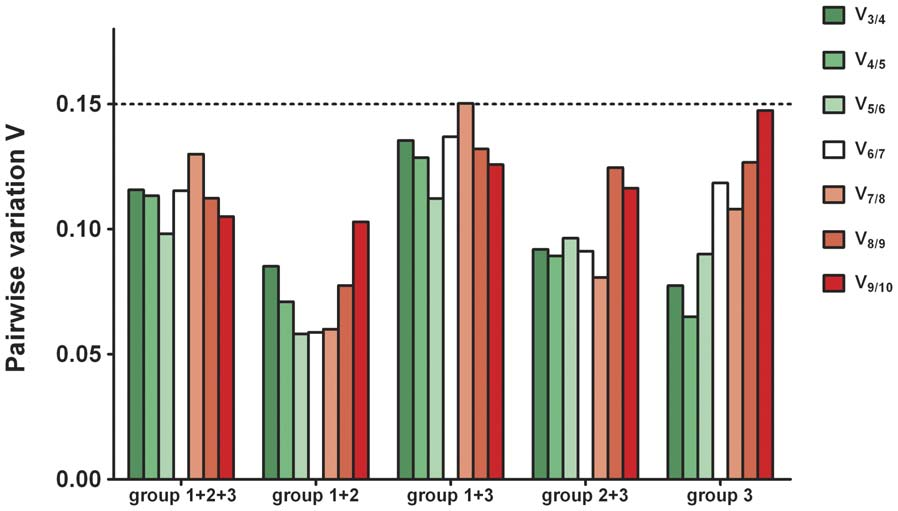
Pairwise variation analysis between sequential normalization factors. The pairwise variation coefficient, calculated between sequential normalization factors, is used to determine of the optimal number of control genes. For instance, V_3/4_ indicates the change in stability when using four instead of three reference genes to calculate the normalization factor. A pairwise variation coefficient of ≤0.15 is regarded as an appropriate cut-off. Therefore, a combination of three reference genes is appropriate to use in each sample subset. To the right, the increasing values are due to the inclusion of unstable reference genes, such as *Actb*, *Polr2a* and *Gapdh* into the analysis. Group 1: infarcted left ventricle; group 2: non-infarcted left ventricle: group 3: sham left ventricular heart tissue.

For each sample, qPCR Taqman® assays were performed for 10 candidate reference genes (table 1). Dilution series were run for all candidate reference genes to quantify qPCR amplification efficiency (table 2). For all genes, samples were assayed within the same PCR-run (sample maximization experimental design) to avoid the introduction of inter-run variation. All samples were analyzed in duplicate. All NTCs were PCR-negative. Relative expression quantities were calculated using the E^−ΔCq^ method. These values were imported in geNorm. Analysis provided a gene expression stability measure (M) for each reference gene that permitted ranking from least stable (highest M value) to most stably expressed (lowest M value) (figure 1). In addition, the pairwise variation value V_n/n+1_ and the effects of stepwise inclusion of the next best reference gene on V_n/n+1_ were calculated (figure 2).

Initially, we performed a geNorm analysis on the overall sample set of 30 infarcted and non-infarcted heart specimens (group 1+2+3), because this is a commonly used experimental setup in mouse myocardial infarction studies. For this experimental setup, *Tpt1*, *Hprt*, *Rpl13a* and *Ppia* showed M values below the theoretical threshold of 0.5, indicating adequate gene stability (figure 1). M values increased moderately for other reference genes, except for *Actb*, *Polr2a* and *Gapdh*, which showed higher gene expression variability as evidenced by an increase in the slope of the M value curve. *Gapdh*, although frequently used for gene expression normalization, yielded the highest M value in this data set, which is indicative of the largest overall variability. In figure 2, the optimal number of reference genes for use in gene normalization can be deduced from the value of the pairwise variation between sequential normalization factors. As recommended by Vandesompele et al. [2] 0.15 is generally considered as an appropriate cut-off, although this should be regarded as a reference rather than a strict value. Below this threshold the addition of extra reference genes does not result in a significant improvement of the expression stability of the reference gene set and is therefor not recommended. In our study, the pairwise variation V_3/4_ between the normalization factors with the combination of the three and four most stable reference genes, yielded a value of 0.117, which was already below the theoretical threshold of 0.15. This means that the addition of *Ppia* to the gene set composed of *Tpt1*, *Hprt* and *Rpl13a* is not needed and only results in a relatively small gain in stability and decrease in pairwise variation. For this reason and because an optimal balance needs to be found between the absolute gain in statistical power by reducing expression variability and the extra costs and efforts when measuring more reference genes, the set of the three best reference genes (NF3) is suitable as reference gene set in our study.

**Figure 3:**
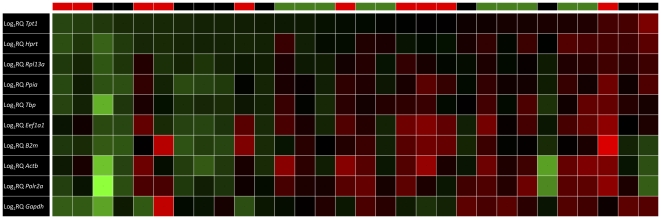
Heatmap of expression variability of individual reference genes. The relative gene expression (RQ) of all samples (n = 30) for the 10 reference genes is depicted (color-coded, expression values are log_2_ transformed). *Tpt1*, *Hprt*, *Rpl13a* and *Ppia* show low variability in gene expression. *Gapdh*, *Polr2a*, *Actb, B2m and Eef1a1* show high within-group expression variability and more extreme - sometimes opposing - expression values for individual samples compared to stably expressed genes. *Tbp* demonstrates intermediate expression variability. Red: ILV; green: NILV; black: sham.

Subsequently, analysis of the tissue subsets revealed that the average expression stability values could be dependent upon the specific tissues used in the experimental setup (figure 1). For analysis of ILV and NILV specimens (group 1+2), all reference genes, except *Gapdh* and *B2m* showed reference gene stability below the theoretical threshold of 0.5. *Tpt1*, *Eef1a1*, *Ppia*, *Rpl13a*, *Hprt* and *B2m* performed best in the sham subset (group 3) and when combining ILV or NILV specimens with sham tissues (group 1+3 or 2+3), M values for *Tpt1*, *Hprt*, *Rpl13a* and *Ppia* or *Rpl13a, Hprt*, *Tpt1*, *Ppia*, *Eef1a1* and *B2m* respectively, were found to meet the aforementioned stability criteria (table 3). In all the tissue subsets the combination of the three best performing genes was found to be sufficient for gene normalization with pairwise variation values of 0.15 or less (figure 2). Interestingly, the *Tpt1*, *Hprt*, *Rpl13a* and *Ppia* genes consistently showed M values below 0.5 in all of the five experimental setups described, while *Eef1a1* displayed acceptable stability three times, *B2m* twice and *Tbp*, *Actb* and *Polr2a* only once. On the other hand, *Gapdh* invariantly disclosed suboptimal gene expression stability in all analyzed subsets. The suboptimal performance of *Gapdh*, *Polr2a* and *Actb*, and to a lesser extent of *B2m*, *Eef1a1* and *Tbp*, was further confirmed by a coefficient of variance analysis using the BestKeeper software (table 4) and by heatmap analysis of the relative gene expression of all reference genes compared accross all samples (figure 3). Also, comparison of the reference gene expression over the three experimental groups showed a manifest (more than twofold) upregulation of *Polr2a*, *Actb*, *B2m* and *Eef1a1* gene expression in ILV or NILV tissues compared to sham controls and a distinct (twofold or more) downregulation of *Gapdh* in ILV specimens compared to sham controls or NILV, respectively. Except for the expression of *Gapdh* in ILV versus sham tissues, all these differential expression patterns, were determined to be statistically significant, even after controlling for false discovery rate (Benjamini-Hochberg correction for multiple comparisons [13]). Selective up- or downregulation of reference genes under specific experimental conditions might, in addition to affecting statistical parameters such as power and sample size, give rise to biased study results when one of these genes would be used as reference for gene expression normalization.

**Table 3:** Reference genes ranked by their expression stability for different sample combinations.

1 + 2 + 3	1 + 2	1 + 3	2 + 3	3
*Gapdh*	*Gapdh*	*Gapdh*	*Polr2a*	*Polr2a*
*Polr2a*	*B2m*	*Polr2a*	*Actb*	*Actb*
*Actb*	***Eef1a1***	*Actb*	*Gapdh*	*Gapdh*
*B2m*	***Actb***	*B2m*	*Tbp*	*Tbp*
*Eef1a1*	***Polr2a***	*Eef1a1*	***B2m***	***B2m***
*Tbp*	***Hprt***	*Tbp*	***Eef1a1***	***Hprt***
***Ppia***	***Tpt1***	***Ppia***	***Ppia***	***Rpl13a***
***Rpl13a***	***Tbp***	***Rpl13a***	***Tpt1***	***Ppia***
***Hprt – Tpt1***	***Ppia – Rpl13a***	***Hprt – Tpt1***	***Hprt – Rpl13a***	***Eef1a1 – Tpt1***

The reference genes are ranked using the expression stability value (M) obtained from the geNorm analysis. M increases from top to bottom. M≤0.5 (bold) indicates stable gene expression. Group 1: infarcted left ventricle; group 2: non-infarcted left ventricle; group 3: sham left ventricular heart tissue.

**Table 4:** Mean, standard deviation (SD) and coefficient of variance (CV) of quantification cycle (Cq) values.

Gene	Cq	SD	CV (%)
*Gapdh*	24.06	1.25	5.19
*Polr2a*	30.85	1.64	5.30
*Actb*	24.46	1.52	6.23
*B2m*	26.24	1.14	4.36
*Eef1a1*	24.97	1.00	4.02
*Tbp*	32.88	1.04	3.15
*Ppia*	27.23	0.82	3.02
*Rpl13a*	26.35	0.67	2.53
*Hprt*	29.99	0.79	2.62
*Tpt1*	25.06	0.66	2.62

CV: coefficient of variance is expressed as the percentage of the Cq standard deviation to the mean Cq.

Finally, to show the effect of suboptimal reference gene selection, we normalized *Nppb* and *Vcam1* expression using different normalization strategies. Both these genes are known to be upregulated in the setting of myocardial infarction [14], [15]. When *Nppb* or *Vcam1* gene expression was normalized against the combination of the three worst performing reference genes (i.e., *Gapdh*, *Polr2a* and *Actb*) or against a combination of all 10 reference genes (NF10), the relative expression differences were blunted and statistical significance was suppressed. However, normalization against the most optimal combination of reference genes (i.e., *Tpt1*, *Hprt* and *Rpl13a*) yielded a higher relative expression difference and a reduction in sample variance, which resulted in a dramatic improvement of the statistical power and significance (table 5).

**Table 5:** Effect of normalization strategy on expression levels and statistical significance.

	Normalization strategy	mean ± SD	mean ± SD	∂	p-value	power
*Nppb*	3 most stable genes	0.65 ± 0.65	-0.43 ± 0.61	1.09	0.003	0.954
	All 10 reference genes	0.49 ± 0.63	0.04 ± 0.85	0.46	0.075	0.252
	3 least stable genes	0.23 ± 0.62	0.35 ± 1.54	-0.12	0.684	0.055
*Vcam1*	3 most stable genes	0.79 ± 0.46	-0.97 ± 0.92	1.76	<0.001	0.999
	All 10 reference genes	0.41 ± 0.44	-0.51 ± 0.61	0.92	0.002	0.953
	3 least stable genes	0.43 ± 0.61	-0.19 ± 0.68	0.62	0.089	0.527

SD: standard deviation; 3 least stable genes: *Gapdh*, *Polr2a*, *Actb*; 3 most stable genes: *Tpt1*, *Hprt* and *Rpl13a*. The mean ± SD are calculated on log_2_ transformed expression data because of non-normality. Two-tailed Mann-Whitney U test was used for calculating p-values. Statistical power was calculated for a Student's t-test using statistical parameters of log_2_ transformed expression data. *Nppb*: group 2 vs. group 3; *Vcam1*: group 1 vs. group 3.

The calculation of the statistical significance and power for the different normalization strategies unambiguously demonstrated that application of suboptimal reference genes (table 6) or gene combinations (table 7), can dramatically affect both these factors. It is clear from this example that inclusion of more reference genes does not necessarily improve the statistical parameters. For example, the combination of the three most stable genes (NF3) required a sample size of 7 or 5 for *Nppb* or *Vcam1,* respectively, in order to obtain a power of at least 0.8. However, normalization using six or more reference genes led to a gradual increase in sample size. In addition, expression analysis using individual reference genes yielded considerable variability in optimal sample size (*Nppb* 5 - 296; *Vcam1* 4 - >1000) and tenfold or more reduction in statistical power (*Nppb* 0.998 - 0.078; *Vcam1* 1 - 0.050).

**Table 6:** Effect of normalization strategy on statistical parameters calculated for *Nppb* and *Vcam1* expression using single reference genes.

		*Tpt1*	*Hprt*	*Rpl13a*	*Ppia*	*Tbp*	*Eef1a1*	*B2m*	*Actb*	*Polr2a*	*Gapdh*
*Nppb*	p-value	<0.001	0.023	0.005	0.023	0.123	0.280	0.631	0.436	0.912	0.005
	power	0.998	0.715	0.933	0.647	0.153	0.231	0.078	0.255	0.107	0.488
	n	**5**	**13**	**7**	**14**	**82**	**48**	**296**	**42**	**137**	**20**
*Vcam1*	p-value	<0.001	<0.001	0.001	0.029	0.004	0.631	0.579	0.105	0.796	0.001
	power	0.998	1	0.992	0.619	0.937	0.136	0.059	0.345	0.050	0.997
	n	**5**	**4**	**6**	**15**	**7**	**96**	**943**	**21**	**>1000**	**5**

Two-tailed Mann-Whitney U test was used for calculating p-values. The statistical power was calculated for a Student's t-test using statistical parameters of log_2_ transformed expression data. Sample size (n) is number per group needed to obtain a power of at least 0.8. *Nppb*: group 2 vs. group 3; *Vcam1*: group 1 vs. group 3.

**Table 7:** Effect of normalization strategy on statistical parameters calculated for *Nppb* and *Vcam1* expression using combinations of multiple reference genes.

		NF3	NF4	NF5	NF6	NF7	NF8	NF9	NF10
*Nppb*	p-value	0.003	0.009	0.007	0.009	0.035	0.029	0.043	0.075
	power	0.954	0.926	0.848	0.748	0.7	0.652	0.417	0.252
	n	**7**	**8**	**9**	**12**	**13**	**14**	**24**	**43**
*Vcam1*	p-value	<0.001	0.001	0.001	0.001	0.003	0.007	0.007	0.002
	power	0.999	0.991	0.993	0.962	0.891	0.835	0.857	0.953
	n	**5**	**6**	**6**	**7**	**9**	**10**	**9**	**7**

Two-tailed Mann-Whitney U test was used for calculating p-values. The statistical power was calculated for a Student's t-test using statistical parameters of log_2_ transformed expression data. Sample size (n) is number per group needed to obtain a power of at least 0.8. *Nppb*: group 2 vs. group 3; *Vcam1*: group 1 vs. group 3.

## Discussion

The importance of adequate reference gene selection for the normalization of qPCR data cannot be underestimated. The inappropriate choice of reference genes frequently results in loss of accuracy, statistical significance and power [16], in particular in case of genes with small expression differences. Over the last decade, several new strategies for data normalization have been proposed and nowadays optimization of reference genes should be recommended as a crucial first step in every gene expression experiment.

Using the geNorm algorithm, we were able to identify a set of three reference genes, *Hprt*, *Rpl13a* and *Tpt1*, which can be used for accurate gene expression normalization in qPCR experiments on mouse myocardial infarction tissue. The application of different combinations of starting material yielded slightly different optimal reference gene sets. However *Gapdh*, which is frequently used for gene expression normalization in many cardiovascular studies, together with *Polr2a* and *Actb,* showed the largest gene variability and the worst performance as reference genes. This finding could be problematic for a substantial number of gene expression studies that utilize *Gapdh* for data normalization. On the other hand, it is not that surprising to find that *Gapdh*, which is an enzyme of the glycolysis pathway, displays high variability in the setting of myocardial infarction, where in the absence of oxygen, anaerobic pathways are activated to fulfill energy demands. Recently, *Gapdh* has also been reported to play a role in the mitochondria during apoptosis, inducing mitochondrial membrane permeabilization, which leads to loss of the inner mitochondrial transmembrane potential [17]. These findings question the unvalidated use of *Gapdh* as a reliable internal reference in ischemic conditions, not only in qPCR experiments, but also for normalization in other molecular techniques, such as western blotting.

The three most stably expressed genes in our experimental setting, *Hprt*, *Rpl13a* and T*p*t1, encode proteins with independent physiological functions. *Tpt1*, also known as histamine-releasing factor (HRF), has a high degree of tissue conservation between species [18] and is expressed in a multitude of different tissues under extensive transcriptional control [19]. It was found to promote cell growth and has anti-apoptotic properties [20]. *Rpl13a* encodes one of about 50 proteins that are part of the large 60S ribosomal subunit, implicating *Rpl13a* in protein synthesis. The *Hprt* gene is translated into the enzyme hypoxanthine-guanine phosphoribosyltransferase, which has a pivotal role in purine nucleotide generation, providing the transfer of a phosphoribosyl group to hypoxanthine or guanine.

Over the last years, only few studies have dealt with reference gene normalization in the setting of myocardial infarction. A first study by Perez et al. [8] described the most suitable control genes in human myocardial tissues. The authors proposed a combination of *Ppia* (encodes a protein folding enzyme of the peptidyl-prolyl cis-trans isomerase family), *Rplp* (a ribosomal subunit protein analogous to *Rpl13a*) and *Gapdh* for accurate normalization. In another study by Pilbrow et al. [6], a microarray screen of publicly available gene expression data was used to select 10 genes of high abundance and consistent gene expression. The subsequent geNorm analysis indicated *Srp14*, *Tpt1* and *Eef1a1* as the most stable genes and ranked *Gapdh* as the least stable gene. The striking difference in *Gapdh* stability between these two studies can be explained by the difference in tissue sampling. The tissues analyzed in the study by Perez et al. [8] were derived from the myocardium of human heart donors not suffering from ischemic heart disease, whereas in the study by Pilbrow et al. [6] specimens from failing hearts together with non-failing donor hearts were analyzed. In our analysis we also noticed a better performance of *Gapdh* in NILV or sham tissues, which supports the observation that *Gapdh* still performed relatively well in the study of Perez et al. [8] On the other hand, the occurrence of contradictory findings between studies with a different study design shows the importance of adequate reference gene analysis in each specific experimental setup, even for different combinations of tissue samples. A recent study investigated human and rodent reference genes in the setting of heart failure and identified *Gapdh* as stable in mouse, but not in rat or human heart tissues [7]. These authors used a data set of only three sham and three heart failure specimens in their analysis. Also, heart failure tissue was harvested at a distance from the infarcted area excluding the infarction border zone and only included the remaining viable septum and left ventricular free wall. As we observed that, in particular in infarcted tissues, *Gapdh* expression behaves very unpredictably compared to other reference genes, the different outcome of this study is probably due to differences in tissue sampling and – importantly – the limited sample size. Our data in the setting of myocardial infarction are in line with the heart failure data of Pilbrow et al. [6] who also found indications for avoiding *Gapdh* as reference gene for routine gene expression normalization in human myocardial studies. However, we have to bear in mind that, because of interspecies differences in gene expression, claims on optimal reference gene combinations are generally not applicable to other species and have to be validated accordingly.

More in general, we stress that our conclusions are valid and refer to myocardial tissues in mice, and more particularly to tissues of a C57BL/6 mouse genetic background. This should be taken into consideration, because strain differences were recently found to result in altered expression stability of reference genes [21]. Secondly, since all tissue specimens were taken one week post-infarction, it is possible that other optimal reference gene combinations might be more appropriate for other timepoints. Furthermore, we limited our analysis to 10 reference genes, which we compiled on the basis of previous reference gene studies in myocardial infarction. We do not exclude the possibility that more optimal reference gene combinations can be found when other stably expressed genes are included in the analysis. However, as evidenced by the low value for the average expression stability value M, our reference gene set outperforms all other reported reference gene sets analyzed so far with the geNorm algorithm in the setting of myocardial infarction. Finally, our findings only apply to the setting of myocardial infarction in mice, and, therefore, do not preclude *Gapdh* from being an adequate reference gene in other conditions, tissues or species.

In conclusion, we identified and validated a stably expressed reference gene set for use in mouse myocardial infarction studies. Optimal reference gene normalization greatly improves statistical significance, power and can dramatically reduce sample size. Our results indicate in particular that *Gapdh*, which is commonly used for gene expression normalization in myocardial infarction studies, has rather high expression variability in myocardial infarction tissues in mice. We furthermore caution against the use of *Gapdh*, *Polr2a*, *Actb*, *B2m* and *Eef1a1* for gene expression normalization in myocardial infarction studies because of selective up- or downregulation after myocardial infarction. Therefore, inclusion of *Gapdh* or other suboptimal reference genes will potentially lead to biased gene expression results. Given the risk of inducing reference gene instability when altering experimental conditions, we recommend the validation of a stable set of reference genes as an initial and essential step in all qPCR experiments.

## Supporting Information

Table S1 **Group characteristics.** The data are shown as mean ± SD. HW: heart weight; BW: body weight; TL: tibia length; LVEDD: left ventricular end-diastolic diameter; AWT: anterior wall thickness. * p<0.05 vs. sham; **p<0.01 vs sham (student's t-test). Transthoracic echocardiography (AplioXV®, 13MHz linear probe, Toshiba) was performed on anesthetized mice one week post-myocardial infarction. Left ventricular end-diastolic diameters (LVEDD) and anterior wall thickness (AWT) were measured at the mid-papillary muscle level.
(DOCX)

Table S2 **MIQE checklist.**
(XLS)
