# Supplementary material for: Importance of Suitable Reference Gene Selection for Quantitative Real-Time PCR: Special Reference to Mouse Myocardial Infarction Studies
Source: PLoS One. 2011 Aug 17;6(8):e23793. doi: 10.1371/journal.pone.0023793 (PMC3157472; doi:10.1371/journal.pone.0023793)
Supplement: Table S1 — Group characteristics. The data are shown as mean ± SD. HW: heart weight; BW: body weight; TL: tibia length; LVEDD: left ventricular end-diastolic diameter; AWT: anterior wall thickness. * p<0.05 vs. sham; **p<0.01 vs sham (student's t-test). Transthoracic echocardiography (AplioXV®, 13MHz linear probe, Toshiba) was performed on anesthetized mice one week post-myocardial infarction. Left ventricular end-diastolic diameters (LVEDD) and anterior wall thickness (AWT) were measured at the mid-papillary muscle level. (DOCX) [file pone.0023793.s001.docx]

**Supplementary Table 1. Group characteristics**

|  |  | **Age (weeks)** | **Weight (g)** | **HW (mg)** | **HW/BW (%)** | **HW/TL (mg/mm)** | **LVEDD (mm)** | **AWT (mm)** |
| --- | --- | --- | --- | --- | --- | --- | --- | --- |
| Group 1 | ILV | 20.5±0.3* | 27.3±3.4 | 154±26* | 0.57±0.12* | 8.40±1.54* | 4.33±0.52* | 0.71±0.13 |
| Group 2 | NILV | 21.9±2.9* | 27.3±2.5 | 143±23 | 0.52±0.07* | 7.90±1.28 | 4.26±0.38* | 0.66±0.16* |
| Group 3 | Sham | 17.7±3.2 | 26.6±2.1 | 126±14 | 0.47±0.03 | 6.99±0.71 | 3.19±0.55 | 0.86±0.19 |
